# Association of digital media use with sleep habits in school children: A cross-sectional study - Author reply

**DOI:** 10.1016/j.sleepx.2025.100143

**Published:** 2025-06-14

**Authors:** Doreswamy Chandranaik, Prawin Kumar

**Affiliations:** Department of Pediatrics, All India Institute of Medical Sciences, Jodhpur, 342005, India

To The Editor,

We thank Shaikh AR et al. [1] for their insightful comments on our earlier published study on digital media use in school children [2]. As we have mentioned, our study was hospital-based, which limits the enrollment of healthy children in the given time frame. It is a misconception that only a large sample size can give a valid result. If the methodology is robust, even a study with smaller sample size can provide valuable information. In addition, a study with a comparable sample size had shown a similar correlation [3]. Moreover, our study contributes practical insights and supports the global findings. Nevertheless, we agree that future studies with larger population-based studies will further strengthen these associations.

Academic performance is ideally assessed using standardized academic records or teacher evaluations. However, due to practical constraints, especially during the post-pandemic recovery phase and limited school access, we employed parental reports as a proxy. This limitation was explicitly acknowledged in our study.

We agree that the objective tools, such as accelerometers**,** can provide more precise and quantifiable measurements of sleep duration and timing in school children [4]. However, the use of such tools was not feasible in our outpatient-based setting due to logistical and resource constraints. Furthermore, despite their subjectivity, validated sleep questionnaires and parental reports are widely used and have demonstrated acceptable reliability for screening purposes, especially in large-scale or resource-limited studies [5]. In the future, the use of affordable wearable equipment will provide more objective assessments in resource-limited settings.

The academic performance is influenced by a multitude of factors beyond digital media usage and could act as confounding factors. However, a study by Sidiqi et al. observed that increased screen time was associated with behavioral problems and lower academic performance, even after adjusting for confounding variables such as sleep and parental supervision. [6]. Our study aimed to explore the association between digital media use and academic performance without implying causality or exclusivity.

In conclusion, digital media use is very common among school children. Many studies, including ours, have highlighted the negative impact of media use on the academic performance in school children. Mass awareness among parents and teachers of its use is the need of the hour.

## CRediT authorship contribution statement

**Doreswamy Chandranaik:** Data curation, Formal analysis, Methodology, Resources, Writing – original draft, Writing – review & editing. **Prawin Kumar:** Conceptualization, Data curation, Formal analysis, Methodology, Supervision, Visualization, Writing – original draft, Writing – review & editing.

## Declaration of competing interest

The authors declare that they have no known competing financial interests or personal relationships that could have appeared to influence the work reported in this paper.
